# More than Just Buying a Van: Lessons Learned from a Mobile Telehealth HCV Testing and Treatment Study

**DOI:** 10.3390/v16091388

**Published:** 2024-08-30

**Authors:** Elyse Bianchet, David de Gijsel, Lizbeth M. Del Toro-Mejias, Thomas J. Stopka, Randall A. Hoskinson, Patrick Dowd, Peter D. Friedmann

**Affiliations:** 1 Office of Research, University of Massachusetts Chan Medical School-Baystate, Springfield, MA 01199, USA; elyse.bianchet@baystatehealth.org (E.B.); lizbeth.deltoro-mejias@baystatehealth.org (L.M.D.T.-M.); randall.hoskinson@baystatehealth.org (R.A.H.J.);; 2 Section of Infectious Diseases, Dartmouth Health, Lebanon, NH 03756, USA; david.degijsel@dartmouth.edu; 3 Better Life Partners, Manchester, NH 03103, USA; 4 Department of Public Health and Community Medicine, Tufts University School of Medicine, Boston, MA 02111, USA; thomas.stopka@tufts.edu

**Keywords:** Hepatitis C virus (HCV), people who inject drugs (PWID), telemedicine, mobile interventions, harm reduction, rural, integrated care

## Abstract

Hepatitis C virus (HCV) disproportionately affects people who inject drugs (PWID). Although HCV has become universally curable since the arrival of direct-acting antivirals, barriers exist to facilitating care and cure in this historically hard-to-reach population, including limited testing and healthcare services and healthcare stigma, issues that are compounded in rural areas. Telehealth is effective in increasing access to HCV care and cure, but innovative approaches of testing and care are required to fully address the need among rural PWID, which led to our study examining a mobile telehealth model for treating HCV. In this commentary, we discuss lessons learned delivering telehealth on a mobile unit, important factors for consideration when designing a mobile intervention, and we suggest an ideal model to increase access to HCV testing and treatment and other services for rural PWID.

## 1. Introduction

The hepatitis C virus (HCV) disproportionally affects people who inject drugs (PWID) [1,2,3,4]. Since the arrival of direct-acting antivirals (DAAs), HCV has become a universally curable infection and a candidate for elimination. This opportunity requires successful engagement of at-risk people to prevent, test, and treat infections [5,6,7,8,9]. PWID are often considered hard to reach and feel stigmatized and underserved by the healthcare system. These barriers to care can be compounded in rural areas of the United States heavily affected by the current opioid crisis [10,11,12].

Various studies have shown the effectiveness of telehealth in increasing access to HCV care and cure [13,14,15,16]. Yet, as a mere alternative to an in-person visit with a clinician, it is still necessary that at-risk people are aware of this care option, trust the clinician and health system, and recognize that they are eligible for treatment regardless of their drug use or sociodemographic characteristics. Facilitated telehealth interventions pair the ease of access to a remote clinician with the relationships and trust built between healthcare teams and patients through community presence, outreach, and patient-centered services [16,17,18]. Mobile interventions have also proven to be effective in reducing barriers to care and have particularly worked alongside harm reduction models like syringe services programs (SSPs). An integrated mobile telehealth HCV treatment model can help to address the high prevalence of both HCV and syringe sharing among many rural PWID [19,20,21,22,23,24].

In this commentary, we share our experience of a mobile telehealth-enhanced HCV treatment study in rural Northern New England in the U.S. In addition to describing the benefits of mobile telehealth, we explore lessons learned and provide recommendations for implementing a mobile program in rural communities that fosters trust, overcomes technical and logistical barriers, and offers opportunities to deliver benefits beyond curing HCV among people who actively use drugs.

## 2. Study Overview

The Drug Injection Surveillance and Care Enhancement for Rural Northern New England (DISCERNNE) study is a federally funded Randomized Controlled Trial examining the effectiveness of a model of mobile telemedicine treatment for HCV integrated with syringe services programming, versus the current clinical practice of referral to a local or regional provider, enhanced with care navigation [25]. A mobile unit, an upfitted Transit cargo van operated by non-clinical research staff and a phlebotomist, traveled between three different rural sites in Vermont and New Hampshire from Spring 2022 to Summer 2024. Rapid antibody testing for HIV and HCV was performed for all enrolled participants (persons with a history of injecting drugs) at the initial screening visit, with confirmatory RNA lab testing for those testing positive for HCV antibodies. Half of eligible participants with active HCV infection were randomized to receive one telehealth visit on the van for prescription of DAAs. Those randomized to the non-intervention group received a referral to community providers enhanced with HCV care navigation. After randomization, a check-in visit and a series of four follow-up visits over the course of approximately one year were completed with participants. Harm reduction services (sterile syringes purchased and supplied by community partner SSPs, safe injection supplies, naloxone, and education) were available for all enrolled participants for the length of the study, including those testing negative for HCV antibodies and RNA (ineligible for randomization). Hepatitis A and B vaccination was also offered to randomized participants lacking immunity.

Data collection and analysis are still ongoing as of publication, so clinical trial findings will not be discussed; a paper publishing results is forthcoming. Rather, this commentary focuses on insights, observations, and recommendations gleaned from our real-world experience, with the intention of presenting information that is broadly generalizable to a variety of organizations and settings interested in enhancing HCV testing and treatment access for a difficult-to-reach, underserved, and at-risk population.

## 3. Mobile Telehealth

In the intervention arm of the study, we paired telehealth with mobile care to offer immediate access to a clinician in a community-based setting, with the van serving as the originating site for a video-assisted encounter. The clinical services were provided by our partner, a healthcare organization focused on providing care for people struggling with addiction, who otherwise deliver care in a hybrid virtual and community-embedded model. All clinical staff, except the phlebotomist on the van, were remote. This integration of telehealth with mobile care provided unique opportunities and challenges.

From the participant’s perspective, the van offered the ability to immediately connect with a clinician from a rural community not usually served by specialists. The van’s internet connection and computer technology (i.e., tablet) obviated the need for a personal video-capable device with reliable internet access and sufficient data allowance.

From a clinician’s perspective, the support provided by the van-based team significantly improved the quality of the encounter. The image and sound quality of the van-based equipment tended to be better than that of patient-owned devices, and the support and troubleshooting of the team on the van compensated for any intrinsic or technological challenges. The semi-formal setting of the van improved focus and dedicated the engagement of participants compared to encounters originating in people’s homes, encampments, or public spaces. Finally, the observations and measurements of the van-based team, such as vital signs and social needs identified in informal conversations, augmented the data gathered in the structured part of the visit.

Scheduling on-demand encounters in real-time and utilizing a pool of clinicians improved efficient use of costly clinician time compared to the fixed cost of a full-time on-site clinician. It helped to avoid losses incurred by no-shows to scheduled appointments and the time spent on referrals and follow-up calls. This efficiency can only be gained if the pool of clinicians is sufficiently large and their alternative clinical work is flexible enough to allow for real-time scheduling. Through our clinical partner, we had access to three to five clinicians at any time, who otherwise provided regular care to the partner’s membership. The increased demand on coordinators and support staff partially offset any potential cost savings from reduced clinician time, although technologic solutions, including instant messaging and dynamic scheduling tools (our partner’s proprietary clinical software) and use of advanced practice providers (e.g., nurse practitioners) instead of physicians can help realize these potential cost savings. The research team pre-scheduled expected visits with clinicians within time windows spanning several hours and provided real-time updates as participants arrived at the van. These pre-established windows of clinician availability also afforded the van team the flexibility to account for participants who were early, late, or whose visits were not able to be planned in advance.

Compared to routine telehealth encounters, a van enables more comprehensive hepatitis care. We were able to vaccinate participants against hepatitis A and B and arrange for laboratory testing when indicated, such as repeat liver tests in participants with isolated HBcAb at increased risk of a hepatitis B flare. Coordinators could facilitate the delivery of DAAs from the pharmacy to the participants, who could pick up their medication at the van if they lacked a reliable delivery address and were not able to go to the pharmacy (most insurers require the use of specialty pharmacies, which are either located in more densely populated areas or solely operate using a mail-order model).

## 4. Staffing the Mobile Unit

Cultivating authentic, compassionate engagement with participants is non-negotiable when working with a population as historically marginalized and stigmatized as people who inject drugs. The importance of consistent, nonjudgmental, genuinely caring staff is not unique to the mobile model, but we found it to be especially indispensable for establishing rapport and community trust quickly. In particular, staff with lived experience, or close personal connections with people with lived experience, facilitated rapport and interpersonal relationships with participants. The variability of schedule and location in a mobile intervention, as opposed to the constancy of a brick-and-mortar site, necessitated effective pathways of communication within the community so that participants could be informed and were able to access services.

In order to collect venous samples for laboratory testing for HCV-RNA and clinical tests required to prescribe DAAs, having an experienced and non-judgmental phlebotomist was an absolute must. Not only is performing phlebotomy difficult in this population, but many people with a history of injection have experienced stigmatization in healthcare, often to a traumatic degree. Given this context, it becomes even more vital that the individual performing the blood draw does so not only with skill, but also the utmost respect, kindness, and attentiveness to the participant’s autonomy and comfort. Therefore, we intentionally sought out and hired compassionate phlebotomists with extensive experience working across diverse clinical settings, including with drug-using populations.

## 5. Partnerships and Parking Site Characteristics

Because our project was a grant-funded study as opposed to a state or locally funded program, we were able to avoid potential issues resulting from permitting and regulatory requirements that can arise when seeking local approval, such as regulations requiring a restroom on board or rules against loitering in public parking areas. This flexibility allowed us to pursue parking the van on the property of privately owned organizations. However, the challenges of finding a partner with a sufficiently sized private parking lot were not insignificant. We faced NIMBYism (not-in-my-back-yard-ism) and denials based on our project population and scope, coupled with a struggle to find a location that was suitably centralized (i.e., close to population centers and other highly utilized services) while also being discreet.

In any community work, good partnerships with local organizations are key, but when the van needs a place to park, they are absolutely necessary. We involved the community partner organizations as early in the planning process as possible to ensure their valuable feedback was taken into account, and care was taken throughout the project to communicate clearly so that any questions or concerns could be addressed. Supportive organizations whose missions and populations aligned, or at least partially overlapped, with the study’s also provided a foundation for recruitment through their established community trust, foot traffic from existing clientele, and the ability to provide mutual referrals. Those organizations should ideally be centrally located and already highly frequented by the target population. Relationships with local SSPs were particularly crucial, as they facilitated the ability to distribute sterile syringes, a necessary service to reduce the risks of HCV and reinfection in those cured. Overall, the organizations we worked with were especially supportive because they recognized our project as addressing a gap in services within their communities and they appreciated the team’s commitment to improving access to care.

## 6. Planning and Logistics

When planning a mobile intervention, real-life considerations must be weighed as heavily as the overall project mission and aims. Logistical preparation must be exhaustive, including considering the facilities that staff and participants will need to use, maximizing storage for supplies (including a compact, portable centrifuge), and deciding on power sources. Planning for private space in the van was essential for telemedicine visits and maintaining participant confidentiality, and access to additional private space in the surrounding area facilitated data collection. In order to maximize discretion and participant confidentiality, it was determined that the van would not be marked with any identifying logos or advertising. The countless logistical details that comprise a “day in the life” on the van cannot be underestimated, adding to the need for ongoing planning, adaptations, and team member flexibility to ensure successful implementation.

For example, in order to decrease reliance on external utilities, which could potentially limit parking options, the van was designed to be self-contained and had no need for external hookups; the sink was fed by an onboard hand-refillable water tank, electricity and climate control were powered by a secondary battery-powered inverter drawing from the vehicle’s battery and engine, and there was no toilet onboard. This increased flexibility, but necessitated access to partner facilities for staff and participants, which was transparently communicated as a key component of any parking arrangement throughout planning discussions.

As noted previously, ideally the parking site would be centrally located, where potential participants already spend time. Staff should also become familiar with where people tend to congregate in order to perform outreach and look for participants when needed for follow-up. Additionally, parking at locations on consistent days and times is very important when working with PWID. Many participants do not have phones or have inconsistent access to one, so a predictable schedule allowed people to find the van and for staff to plan future check-ins and return visits. Consistency also ensures that word-of-mouth referrals lead to connections with new people interested in getting tested or treated for HCV. However, we ultimately found that a balance between having set locations and being hypermobile, i.e., able to drive to people unable to reach fixed sites but who were able to contact study staff via phone or WiFi messaging apps, was necessary, helping to overcome individual barriers to van access (e.g., no transportation, participant banned from a partner organization).

That balance, though, was delicate to strike at times, based on current staffing or real-time volume; for instance, completing ongoing or already scheduled visits at the fixed site would take precedence. Also, while driving the van to meet someone facilitated an interaction with that individual, doing so potentially compromised the ability to serve any number of people who may have been expecting to access the van at the fixed site (e.g., participants knew the van parked at the shelter on Tuesdays and could stop by for check-ins or supplies on that day). Thus, sticking to the consistent schedule always took priority. If staffing allowed, staff may have been dispatched to do visits, but certain research activities, such as telehealth and phlebotomy, had to be performed on the van per study protocol and health and safety standards. If hypermobility was required, and dispatching a staff member was not an option due to short staffing, existing appointments, or time constraints, efforts were made to drive the van to another location at the end or the beginning of the field day so that the established parking schedule was adhered to as closely as possible.

## 7. Mobile vs. “Actually Mobile”: Brainstorming the Ideal Model to Increase Access to Telehealth and Other Services for Rural PWID

People are mobile, while resources, time, and staffing are limited. A mobile telehealth model allows an organization to maximize not only service volume, but also geographic coverage, a uniquely vital component in rural areas. In our experience, the benefits of this model were further maximized when we had both a fixed parking schedule as well as the ability to be hypermobile—or “actually mobile”, as we often joked—driving the van to an individual or sending a staff member to meet with participants in a separate vehicle.

Thus, our experience suggests that the most effective way to serve rural PWID and expand access to HCV testing and treatment, as well as telehealth and other services, is to utilize a hybrid mobile–hypermobile approach. Due to a lack of transportation, a geographically dispersed environment, and lower population densities in rural areas, it is highly advantageous to have the ability to move to alternating fixed sites based on community needs. That said, the parking schedule should be consistent enough that services are accessible to people without phones or internet access; responsiveness is an incredible asset, but mobile staff cannot respond to requests from those who have no way to initiate contact. Thus, the ideal approach would include a set location as well as the ability to dispatch another vehicle from that mobile location, or have other staff separately providing outreach, medication delivery, and harm reduction services on-demand. If limited by staffing or capacity, parking at fixed sites and performing on-demand response would alternate days on a set schedule.

In sum, a successful rural service delivery paradigm would benefit from a three-pronged approach: a brick-and-mortar site, or homebase, where participants have access to basic services and amenities; a mobile unit, traveling regularly to rural communities with limited access to services; and an on-demand, responsive, dispatched vehicle, moving through the rural landscape to give people what they need and deserve, when they need it. If services are not designed with the hardest-to-reach populations in mind, the prevention and ultimate elimination of HCV, too, will remain out of reach.

## Data Availability

Not applicable.
